# The Rubber Patent Case

**Published:** 1872-05

**Authors:** 


### The Rubber Patent Case.

In the case of the Rubber Company against Dr. B. E. Gardner, and brought by appeal before the Supreme Court of the United States, the arguments were made in March last, and elicited the unqualified approbation of all who heard them. The decision has not yet been rendered, but is looked for daily. This decision is anxiously looked for by a large part of the profession, as it is a matter of special interest now that the Goodyear patent expires in a few days; it will either release dentists from any embarrassment in the use of rubber for the construction of artificial dentures, or put a restraint upon its use for some nine years yet.

